# Analysis of clinical characteristics and bronchoalveolar lavage fluid microbial community diversity in non-cystic fibrosis bronchiectasis patients with *Pseudomonas aeruginosa* colonization

**DOI:** 10.1128/spectrum.00871-26

**Published:** 2026-06-23

**Authors:** Tingxiu Peng, Chao Liu, Ping Li, Xiaojian Xiong, Yujie Wang, Ping Zhang, Junyao Li, Qin Zhang, Wei Zhang, Ying Ying

**Affiliations:** 1 Jiangxi Provincial Key Laboratory of Respiratory Diseases, Jiangxi Institute of Respiratory Diseases, The Department of Respiratory and Critical Care Medicine, The First Affiliated Hospital, Jiangxi Medical College, Nanchang University 117970, Nanchang, Jiangxi, People's Republic of China; 2 Jiangxi Clinical Research Center for Respiratory Diseases, Nanchang, Jiangxi, China; 3 China-Japan Friendship Jiangxi Hospital, National Regional Center for Respiratory Medicine, Nanchang, Jiangxi, China; 4 Jiangxi Provincial Key Laboratory of Prevention and Treatment of Infectious Diseases, Jiangxi Medical Center for Critical Public Health Events, The First Affiliated Hospital, Jiangxi Medical College, Nanchang University 74653, Nanchang, Jiangxi, People's Republic of China; 5 Emergency Trauma Center, The First Affiliated Hospital, Jiangxi Medical College, Nanchang University 74653, Nanchang, Jiangxi, China; Nova Southeastern University, Fort Lauderdale, Florida, USA

**Keywords:** *Pseudomonas aeruginosa*, non-cystic fibrosis bronchiectasis, bronchoalveolar lavage fluid, 16S rRNA sequencing

## Abstract

This study aims to examine the differences in microbial community abundance and species distribution in the bronchoalveolar lavage fluid (BALF) obtained from non-cystic fibrosis bronchiectasis (NCFB) patients between those with and without *Pseudomonas aeruginosa* (*P. aeruginosa*) colonization, and to assess the impact of *P. aeruginosa* colonization on airway microecological imbalance in NCFB. Sixty-four patients were enrolled, grouped into *P. aeruginosa*-colonized (PA, 29) and non-colonized (NPA, 35) groups. Among patients with NCFB, those with *P. aeruginosa* colonization had greater disease severity than those without colonization, with significantly higher bronchiectasis severity index (BSI) scores (*P* < 0.05). The proportions of patients with higher Bhalla and E-FACED scores were also greater in the PA group than in the NPA group (62.1% vs 42.8% and 34.1% vs 20%, respectively). Annual hospitalization frequency was numerically higher in PA (2.14 ± 2.43 vs 1.47 ± 0.86; *P* = 0.543). The PA group exhibited poorer pulmonary function, with significantly lower FEV1 and FEV1% predicted (both *P* < 0.01). In addition, NCFB patients in the PA group showed higher proportions of elevated white blood cell counts (31% vs 17.1%) and neutrophil percentages (41.4% vs 31.4%). Microbiota analysis of BALF demonstrated reduced alpha diversity in NCFB patients with *P. aeruginosa* colonization, with a predominance of *Pseudomonas*, whereas non-colonized NCFB patients had relatively higher abundances of *Streptococcus*, *Acinetobacter*, *Rothia*, *Veillonella*, and *Prevotella*. Overall, *P. aeruginosa* colonization in NCFB is associated with increased disease severity, heightened inflammation, and impaired lung function, accompanied by decreased microbial diversity, *Pseudomonas* dominance, and suppression of commensal taxa.

**IMPORTANCE:**

*Pseudomonas aeruginosa* is a common colonizer in the airways of patients with non-cystic fibrosis bronchiectasis (NCFB), linked to disease severity, but research on its impact on clinical outcomes and airway microbiome diversity remains limited. This study compared *P. aeruginosa*-colonized (PA group) and non-colonized (NPA group) NCFB patients and found that the PA group had more severe disease (higher exacerbation, severity scores) and reduced bronchoalveolar lavage fluid (BALF) microbial α/β diversity (marked by *Pseudomonas* dominance and suppressed symbionts like *Streptococcus*). These findings indicate that *P. aeruginosa* reshapes the microecology and is associated with airway microbial imbalance. This study confirms that *P. aeruginosa* colonization is a key factor in NCFB disease progression and airway microecological imbalance, highlighting reduced colonization and restored homeostasis as precision intervention strategies that inform targeted therapies.

## INTRODUCTION

Bronchiectasis is a chronic respiratory disease characterized by abnormal bronchial dilation and chronic inflammation, associated with various infectious, immune, autoimmune, allergic, and genetic disorders. Its core pathological mechanism is a vicious cycle of recurrent airway infections and inflammatory responses (1, 2). Non-cystic fibrosis bronchiectasis (NCFB), the most prevalent type of bronchiectasis, is characterized by chronic respiratory symptoms and radiographic airway dilation (3). Unlike cystic fibrosis (CF), NCFB has diverse etiologies, including post-infectious injury, immunodeficiency, and genetic abnormalities; however, infection with *Pseudomonas aeruginosa* is one of its most prominent clinical features (4).

*P. aeruginosa* is the most common chronic colonizer in NCFB patients, significantly associated with disease severity (e.g., bronchiectasis severity index [BSI], Bhalla score), acute exacerbation frequency (exacerbations/year), and long-term prognosis (all-cause mortality, lung function decline rate) (5, 6). The BSI (7) and E-FACED score (8) are clinical tools that identify patients at risk of death, hospitalization, and deterioration, which are closely linked to disease severity. Studies show that higher BSI/E-FACED scores correlate with greater severity, manifesting as increased exacerbations, hospitalizations, and mortality (9, 10). Epidemiological data indicate that *P. aeruginosa*-colonized patients have worse phenotypes: increased sputum production, higher lobar involvement, dyspnea, impaired pulmonary function (reduced FEV₁% pred, FEV₁/FVC), and elevated risk of exacerbation-related hospitalization (5, 11, 12). Chronic purulent sputum and dyspnea also impair quality of life, prolonging compromised well-being (13).

*P. aeruginosa* pathogenicity involves multifactorial synergy: it secretes elastase and pyocyanin, compromising the airway epithelial barrier, thickening mucus, impairing cilia, and causing mucus retention and neutrophil infiltration (14). These factors also damage bronchial elastic fibers, accelerating irreversible dilation. Additionally, its multidrug resistance (e.g., β-lactams/fluoroquinolones), biofilm formation (15), and mutation/deletion adaptation to the lung environment (16) enable persistent airway colonization, challenging anti-infective therapy. Recent studies show *P. aeruginosa* invades goblet cells, breaches epithelium, disrupts integrity, and increases bacterial translocation/dissemination to basal epithelium (17).

*P. aeruginosa* colonization is closely associated with the severity of disease and the diversity of airway microbiota in patients with NCFB: on one hand, the airway microenvironment affects the colonization process of *P. aeruginosa*; on the other hand, after *P. aeruginosa* colonizes, it can also further regulate the activity of other microorganisms by altering the airway environment (such as secreting virulence factors and competing for nutrients). This bidirectional interaction may jointly affect the survival and colonization ability of *P. aeruginosa* in the airways of NCFB patients (18, 19). *P. aeruginosa* colonization often leads to a decrease in the abundance of other microorganisms, and its mechanism may be related to its multidrug resistance. Under conditions of combined drug use, highly resistant *P. aeruginosa* can survive, while other sensitive bacterial populations are selectively killed. *P. aeruginosa* takes this opportunity to continuously evolve and maintain its colonization state (20). Currently, research on the impact of *P. aeruginosa* colonization on clinical symptoms and airway microbiota in NCFB patients remains limited by small sample sizes, neglect of strain heterogeneity, and insufficient optimization of treatment strategies. Additional clinical data are needed to serve as the clinical basis for subsequent mechanistic studies.

This study aims to analyze the impact of *P. aeruginosa* colonization in NCFB on epidemiological characteristics, clinical impacts, and microbial community composition, and further provides clinical and bronchoalveolar lavage fluid (BALF) sequencing data analysis on the effects of *P. aeruginosa* colonization on the clinical characteristics and airway microbiota of NCFB patients, offering data support for a deeper understanding of the pathogenic mechanisms of *P. aeruginosa* colonization, thereby providing a scientific basis for optimizing NCFB management. We found that patients with NCFB colonized by *P. aeruginosa* had higher disease severity scores compared to non-colonized patients, and the airway microbial abundance was reduced in patients colonized with *P. aeruginosa*.

## MATERIALS AND METHODS

### Collection of clinical data

Clinical data were collected from patients with NCFB diagnosed at The First Affiliated Hospital of Nanchang University from 1 February 2025 to 31 July 2025. The data included basic information, such as name, gender, age, comorbidities, laboratory test results, imaging reports, and pulmonary function tests. The study enrolled hospitalized NCFB patients and classified them into two groups based on BALF and sputum culture results: the *P. aeruginosa* colonization group (PA group, defined as positive for *P. aeruginosa* in BALF or sputum culture) and the non-colonized group (NPA group, defined as no *P. aeruginosa* detected). Diagnosis of NCFB was based on the Expert consensus on the diagnosis and treatment of adult bronchiectasis in China (2021 Edition) (21). Airway colonization with *P. aeruginosa* was defined as a positive result in sputum or BALF bacterial culture during clinical stability (no acute exacerbations and no systemic antibiotic therapy within at least 4 weeks). While international guidelines and studies by Pieters et al. define chronic colonization based on at least two positive cultures months apart (22), we used a single positive culture during clinical stability to identify current airway colonization. According to the routine reports from our hospital’s clinical microbiology laboratory, the positivity threshold for *P. aeruginosa* was set at ≥10^4^ CFU/mL; samples with concentrations below this cutoff were classified as *P. aeruginosa*-negative.

#### Inclusion criteria

- Age ≥ 18 years.
- Patients with NCFB meeting the diagnostic criteria of the Chinese Expert Consensus on Diagnosis and Treatment of Adult Bronchiectasis (2021 Edition).
- Availability of a qualified BALF sample (defined as no obvious bloody contamination, total cell count ≥10⁵ cells/mL, and standardized bacterial culture procedures).
- Positive result for *P. aeruginosa* in BALF bacterial culture.

#### Exclusion criteria

- Loss to follow-up during the study period (failure to complete the collection of all clinical data or follow-up).
- Presence of any of the following conditions: active tuberculosis, lung cancer, primary immunodeficiency diseases (e.g., cystic fibrosis, primary ciliary dyskinesia), severe immunosuppressive states (e.g., long-term use of glucocorticoids/immunosuppressants, HIV infection), or other pulmonary diseases that may affect the airway microbiome (e.g., lung abscess, diffuse panbronchiolitis).
- Acute asthma exacerbation at enrollment or history of acute asthma exacerbation within the past 3 months.

Clinical data were extracted from the electronic medical records of hospitalized patients. The data included demographic characteristics (age, gender), comorbidities (such as hypertension, diabetes mellitus, chronic obstructive pulmonary disease [COPD], asthma), length of hospitalization, laboratory test results, and microbiological results. Additional patient information was collected via telephone follow-up and outpatient visits until 31 July 2025, or until loss to follow-up or death.

### Collection and processing of bronchoalveolar lavage fluid samples

Approximately 10 mL of lavage fluid was collected from the target lobe (affected area determined by high-resolution CT or clinical evaluation) via bronchoalveolar lavage. Immediately after collection, the mixed BALF sample was placed in a pre-cooled ice box (4°C) to prevent DNA degradation and promptly transported to the laboratory within 1 h. At 4°C, the sample was centrifuged at 300 × *g* for 10 min, and the pellet (containing microorganisms) was transferred to a new centrifuge tube. The pellet was resuspended in phosphate-buffered saline (PBS, pH 7.4) at a 1:1 volume ratio and stored in a −80°C ultra-low temperature freezer for subsequent microbiome analysis.

### DNA extraction and 16S rRNA gene amplification

DNA was extracted using a commercial kit (Qiagen DNeasy Blood and Tissue Kit, 69504). Using the extracted DNA as a template, PCR amplification was performed to target the V3–V4 hypervariable region of the 16S rRNA gene. Primer sequences were as follows: forward (F): 5′-ACTCCTACGGGAGGCAGCA-3′; reverse (R): 5′-GGACTACHVGGGTWTCTAAT-3′.

Library preparation and sequencing were performed on the Illumina NovaSeq 6000 platform (paired-end, 2 × 250 bp) by TsingkeBiotechnology Co., Ltd. (Beijing, China). Raw reads obtained from sequencing were filtered using Trimmomatic (23) software, followed by primer sequence removal with Cutadapt (24) software to generate clean reads. Paired-end sequences were processed using the DADA2 (25) plugin in QIIME2 (26) to infer amplicon sequence variants (ASVs). This pipeline involved denoising to correct sequencing errors, merging paired-end reads, and removing chimeric sequences, ultimately yielding a high-quality, non-chimeric data set. Raw data were uploaded to the BaseSpace cloud platform (Illumina, USA) for initial quality control (visualization of quality distribution and adapter content). Subsequent species annotation (using the SILVA 138.1 database) and diversity analysis (α/β diversity) were completed on a local server to ensure data traceability and analytical reproducibility. Regarding sequencing depth, 64 samples were sequenced for this project. After stringent quality filtering and denoising with the DADA2 pipeline, we obtained 4,378,346 high-quality Clean Reads. The sequencing depth was robust, with a minimum of 41,545 Clean Reads per sample and an average of 68,411 Clean Reads per sample. This depth far exceeds the recommended threshold for 16S rRNA amplicon sequencing, ensuring saturated coverage of the microbial community and reliable detection of low-abundance taxa. The raw sequence data are available in the Sequence Read Archive (SRA) at PRJNA1438598.

### Statistical analysis

Statistical analysis was performed using SPSS 25.0. For continuous variables, the Shapiro-Wilk test was used to assess the normality of data distribution. Data conforming to a normal distribution were presented as mean ± SD, and group comparisons were conducted using the independent samples *t*-test. In contrast, data not conforming to a normal distribution were presented as median (interquartile range, IQR) [M (P25, P75)], and group comparisons were performed using the Mann-Whitney *U* test.

For categorical variables, the chi-square test was applied. First, the theoretical frequencies (T) of all cells in the contingency table were calculated. The specific test selection was as follows: if all cells had T ≥ 5, the Pearson chi-square test was used; if 1 ≤ T < 5 in any cell, the continuity-corrected chi-square test was applied; if T < 1 in any cell or the total sample size was too small, Fisher’s exact test was employed. *P* < 0.05 was considered statistically significant in two-tailed tests.

## RESULTS

### Clinical characteristics of enrolled NCFB patients

While *P. aeruginosa* colonization is implicated in NCFB progression, its specific impact on clinical phenotypes remains unclear. To address this, we prospectively enrolled 64 clinically stable NCFB patients and stratified them by colonization status (Table 1). We compared baseline demographic and clinical characteristics between the *P. aeruginosa*-colonized group (PA, *n* = 29) and the non-colonized group (NPA, *n* = 35).

**TABLE 1:** Comparison of clinical characteristics of NCFB patients with or without *P. aeruginosa* colonization^*a*^

Clinical characteristics	PA (*n* = 29)	NPA (*n* = 35)	*t*/*Z*/χ^2^	*P* value
Age (years)	61 (50.5, 68)	67 (52, 74)	−1.275	0.202
Gender (male)	19 (65.5%)	17 (48.6%)	1.851	0.174
Length of hospital stay	10 (7, 13)	8 (6, 11)	−1.882	0.060
Annual number of hospitalizations	2.14 ± 2.43	1.47 ± 0.86	−0.609	0.543
Smoking	7 (24.1%)	5 (14.3%)	1.010	0.315
BSI score				
Mild (0–4)	0 (0%)	20 (57.1%)	−4.871	<0.001
Moderate (5–8)	17 (58.6%)	9 (25.7%)	−2.647	0.008
Severe (≥9)	12 (41.4%)	6 (17.1%)	−2.130	0.033
Bhalla score				
Mild (0–8)	11 (37.9%)	20 (57.1%)	−1.519	0.129
Moderate (9–16)	16 (55.2%)	13 (37.1%)	−1.431	0.152
Severe (17–25)	2 (6.9%)	2 (5.7%)	−0.193	0.847
E-FACED score				
Mild (0–3)	19 (65.5%)	28 (80%)	−1.296	0.195
Moderate (4–6)	9 (31%)	7 (20%)	−1.007	0.314
Severe (7–9)	1 (3.5%)	0 (0%)	−1.099	0.272
Blood cell count				
WBC (×10^9^/L)	8.53 ± 3.45	7.62 ± 2.72	1.128	0.264
Elevated WBC	9 (31%)	6 (17.1%)	1.706	0.192
Neutrophil (%)	77.55 (69.78, 82.78)	73.30 (65.1, 76)	−0.382	0.787
Elevated neutrophil (%)	12 (41.4%)	11 (31.4%)	0.682	0.409
Monocyte (%)	6.46 ± 4.1	6.73 ± 1.89	−0.321	0.750
C-reactive protein	22.13 (5.21, 113.23)	5.95 (3.45, 57.87)	−1.222	0.222
ESR	44.88 ± 32.06	41.33 ± 20.75	0.235	0.818
BALF cell count				
Macrophage (%)	5 (3, 8)	13 (3.5, 31.5)	−1.501	0.133
Neutrophil (%)	90 (85, 93)	82 (56.5, 93)	−1.571	0.118
Lymphocyte (%)	3 (3, 5)	5 (3, 6.5)	−0.902	0.367
Eosinophil (%)	0 (0, 1)	0 (0, 1)	−0.283	0.777
Pulmonary function				
FEV_1_ (L)	1.31 (0.86, 1.82)	1.98 (1.54, 2.37)	−3.055	0.002
FEV_1_%pred (%)	64 (45, 81.85)	85 (75, 93)	−3.001	0.003
FEV_1_/FVC (%)	78.1 ± 14.77	82.29 ± 14.39	−1.143	0.257
Comorbidities				
Hypertension	7 (24.1%)	4 (11.4%)	1.018	0.313
Diabetes mellitus	7 (24.1%)	1 (2.9%)	4.765	0.029
Asthma	4 (13.8%)	0 (0%)	3.064	0.080
COPD	10 (34.5%)	9 (25.7%)	0.584	0.445
OPT	5 (17.2%)	4 (11.4%)	0.093	0.761
Chronic hepatitis B	5 (17.2%)	4 (11.4%)	0.093	0.761

^*a*^ BSI score, bronchiectasis severity index (BSI) scoring form; WBC, white blood cells; ESR, erythrocyte sedimentation rate; FEV₁, forced expiratory volume in 1 s; FEV₁%pred, percentage of FEV₁ predicted; FEV₁/FVC, ratio of FEV₁ to forced vital capacity (FVC); BALF, bronchoalveolar lavage fluid; OPT, old pulmonary tuberculosis; COPD, chronic obstructive pulmonary disease; *P* < 0.05 was considered statistically significant.

Comparative analysis revealed that the cohorts were well-matched for key demographic variables, including gender distribution, age, length of hospital stay, annual hospitalization frequency, and smoking history (all *P* > 0.05) (Table 1). Despite this demographic homogeneity, we observed a distinct biological trend toward increased disease severity in the PA group.

#### Disease severity scores

To determine whether *P. aeruginosa* colonization is associated with a distinct clinical phenotype, we first compared the baseline demographics and disease severity scores between the PA and NPA groups. While both cohorts were demographically similar (Table 1), we observed a significant shift in disease severity distribution, favoring more advanced stages in the PA group. Analysis using the BSI revealed a marked skew toward higher scores in colonized patients (Table 1). Specifically, severe BSI scores (≥9 points) were observed in 41.4% of PA patients compared to only 17.1% in the NPA group (*P* = 0.033), while mild cases (0–4 points) were almost exclusively found in the NPA cohort (57.1% vs 0%, *P* < 0.001). This confirms that *P. aeruginosa* colonization is strongly linked to a more severe clinical trajectory.

In contrast, when assessing structural lung damage using the Bhalla and E-FACED scoring systems, we found no statistically significant overall differences between the groups. However, descriptive analysis revealed a consistent biological trend: the proportion of patients with moderate-to-severe Bhalla scores was higher in the PA group (62.1% vs 42.8%), as was the proportion of moderate-to-severe E-FACED scores (34.1% vs 20%). These data suggest that while radiographic scoring may not fully capture the functional impact of colonization, the trend toward worse structural outcomes persists.

#### Systemic and airway inflammatory profiles

Given the role of systemic inflammation in NCFB progression, we compared peripheral blood leukocyte counts and neutrophil percentages between PA and NPA groups. While a higher proportion of colonized patients exhibited elevated white blood cell (WBC) counts (31% vs 17.1%) and increased neutrophil percentages (41.4% vs 31.4%), these differences failed to reach statistical significance (WBC: *P* = 0.192; neutrophil percentages: *P* = 0.409). Although underpowered to confirm statistical separation, the numerical trend toward heightened systemic inflammation in the PA group aligns with the observed disease severity profiles.

Regarding localized airway inflammation, differential cell counts were performed on BALF. Analysis revealed that the median neutrophil percentage was higher in the PA group for 90% (85%, 93%) than in the NPA group for 82% (56.5%, 93%). Biologically, the PA group demonstrated a strikingly tight clustering of high neutrophil counts, with the middle 50% of patients confined to a narrow range of 85%–93%. In contrast, the NPA group exhibited a much broader and more variable distribution (56.5%–93%). While statistical significance was not achieved, this descriptive pattern suggests that intense neutrophilic infiltration may be a more consistent feature within the *P. aeruginosa*-colonized airway, whereas non-colonized patients display heterogeneous inflammatory patterns.

#### Pulmonary function

In order to objectively assess the impact of *P. aeruginosa* colonization on respiratory physiology, we compared pulmonary function parameters between the PA and NPA groups. Our analysis focused on airflow limitation and obstruction indices. We found that colonized patients exhibited significantly compromised lung function. The PA group had a lower median forced expiratory volume in 1 s (FEV1) of 1.31 L (0.86, 1.82) compared to 1.98 L (1.54, 2.37) in the NPA group (*P* = 0.002). This reduction was also evident in normalized values, with FEV1% predicted significantly lower in the PA group, 64% (45, 81.85%), than in the NPA group, 85% (75, 93%) (*P* = 0.003). Although the difference in the FEV1/FVC ratio was not significant (*P* = 0.257), the FEV1/FVC ratio in the PA group showed a trend of being lower than in the NPA group (78.1 ± 14.77 vs 82.29 ± 14.39). These results suggest that while *P. aeruginosa* colonization is associated with more severe airflow obstruction, it does not disproportionately affect the vital capacity ratio relative to the overall obstruction severity.

#### Distribution of comorbidities

We analyzed the prevalence of common concomitant diseases in both cohorts. While most conditions occurred at similar frequencies, we observed a distinct difference in the prevalence of diabetes mellitus. Diabetes was significantly more prevalent in the PA group (24.1%, 7/29) compared to the NPA group (2.9%, 1/35; *P* = 0.029). Other comorbidities, including hypertension (24.1% vs 11.4%), asthma (13.8% vs 0%), COPD (34.5% vs 25.7%), old pulmonary tuberculosis (17.2% vs 11.4%), and chronic hepatitis B (17.2% vs 11.4%), were numerically higher in the PA group but did not reach statistical significance. These data suggest that diabetes mellitus is specifically associated with the *P. aeruginosa*-colonized phenotype in NCFB patients, whereas other comorbidities are likely distributed randomly between groups.

### Impact of *P. aeruginosa* airway microbial diversity

#### Bioinformatics and statistical analysis

**Fig 1:**
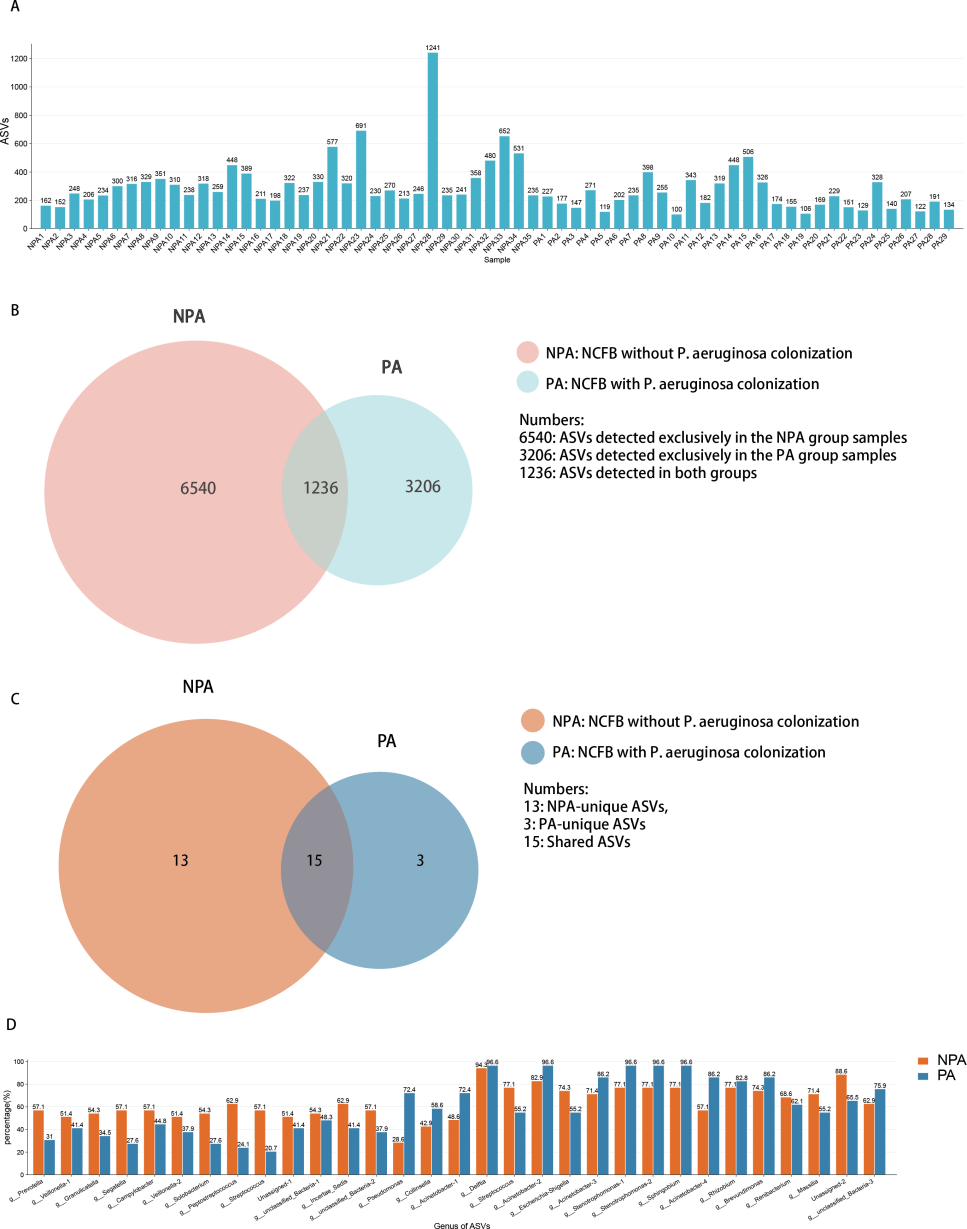
ASV of species. (**A**) Distribution map of ASV counts, with sample names on the x-axis and the number of ASVs on the y-axis. (**B**) Venn diagram illustrating the detected ASVs in the PA and NPA group samples and the number of ASVs detected in both groups. (**C**) Venn diagram illustrating the unique and shared ASV numbers among samples or groups. (**D**) Specific distribution of ASVs unique and shared between PA and NPA groups; orange represents the NPA group, blue represents the PA group.

A total of 64 BALF samples from the PA group and the NPA group were subjected to 16S rRNA sequencing, yielding a total of 4,946,619 raw reads. After ASVs clustering and taxonomic annotation, Venn diagrams were generated. Statistics showed that the 64 samples contained 10,982 ASVs (Fig. 1A). Initially, using a lenient definition, we identified 3,206 ASVs detected in the PA group and 6,540 detected in the NPA group (Fig. 1B). However, recognizing that such metrics may capture stochastic detection in single samples, we applied a prevalence-based threshold: an ASV was considered group-specific only if present in ≥50% of samples within one group and <50% within the other. This stringent re-analysis drastically reduced the number of unique features, identifying only 3 PA-unique ASVs and 13 NPA-unique ASVs, with 15 ASVs shared between groups (Fig. 1C and D).

To exclude the possibility of contamination from DNA extraction kit, we performed LabChip fragment analysis on the sequencing libraries. The assay included Lower Markers (LM, ~1 bp) and Upper Markers (UM, ~10,000 bp) to calibrate the electrophoresis. No amplification products were detected in the kit-only negative controls within the 1–10,000 bp range (Fig. S1A), confirming that the *Pseudomonas* sequences detected were not derived from reagent contaminants. All clinical samples displayed specific amplification peaks within the expected fragment size range. The amplicon size distribution and sequencing read data indicated that the majority of reads in both groups were concentrated within the optimal fragment size range (430–440 bp), confirming the success and specificity of the library preparation (Fig. S1B). This confirms that the detected Pseudomonas sequences represent genuine biological signals rather than environmental contaminants.

#### Impact of *P. aeruginosa* colonization on airway microbial alpha diversity

**Fig 2:**
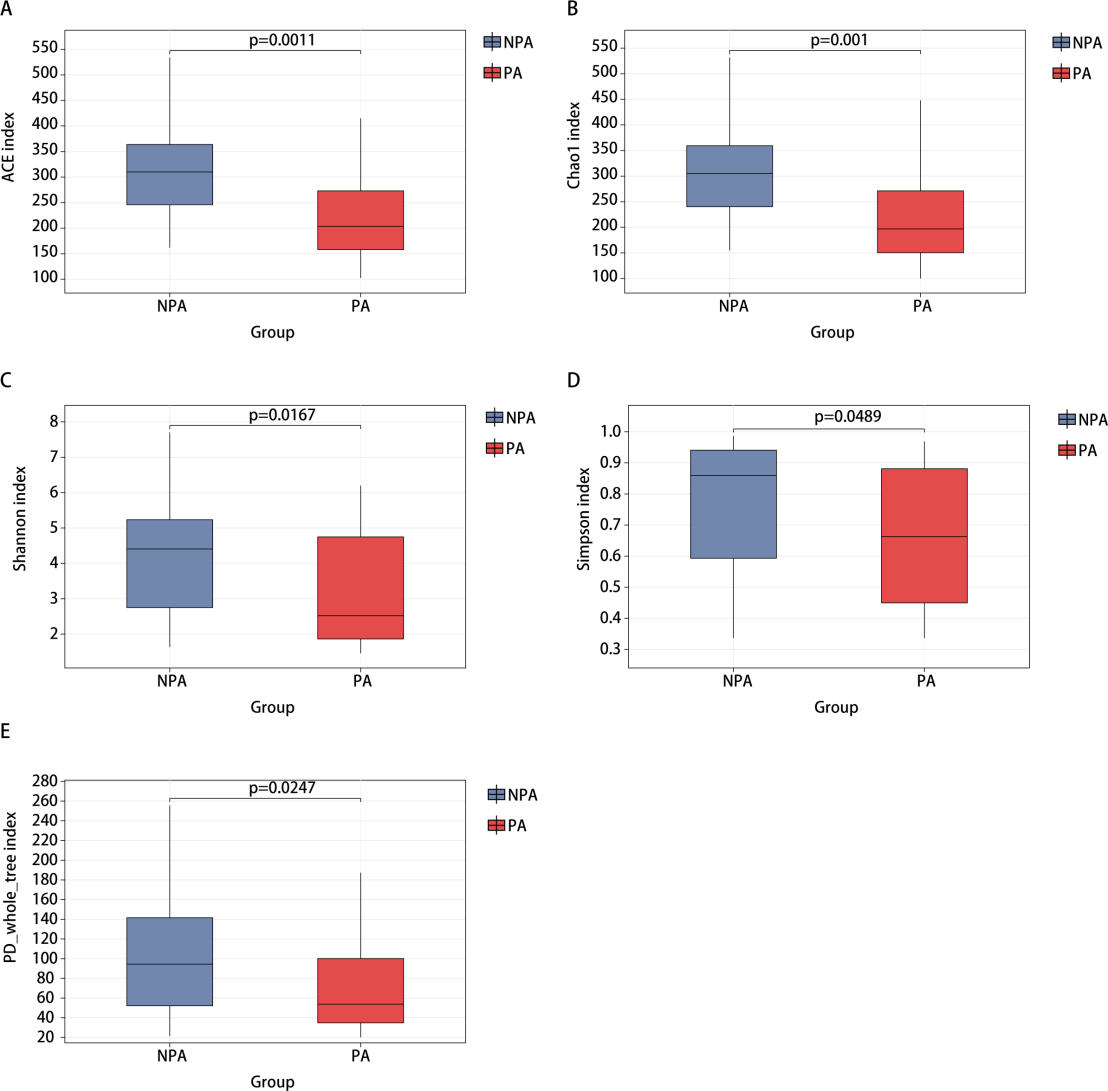
Analysis of alpha diversity indices. (**A–E**) ACE: abundance‐based coverage estimator, an index estimating the total number of species based on the species accumulation curve; Chao1: a community species richness index based on ASVs; Shannon: a comprehensive diversity index reflecting both species richness and evenness; Simpson: a diversity index reflecting the proportion of dominant species; PD_whole_tree: a community phylogenetic diversity index based on the phylogenetic tree. Note: the horizontal axis represents different groups, and the vertical axis represents the alpha diversity index values. The Wilcoxon test was used, and *P* < 0.05 indicates a statistically significant difference.

Characterizing the alpha diversity profiles of the airway microbiota revealed a profound loss of diversity in NCFB patients with *P. aeruginosa* colonized. We characterized the alpha diversity profiles of the airway microbiota in PA and NPA groups. Analysis of five core diversity indices, including ACE, Chao1, Shannon, Simpson, and PD_whole_tree, revealed a consistent and significant reduction in the PA group compared to the NPA group (*P* < 0.05 or *P* < 0.001; Fig. 2A through E). This comprehensive decrease indicates that *P. aeruginosa* colonization is associated with a collapse in species richness, community evenness, and phylogenetic diversity within the airway ecosystem.

**Fig 3:**
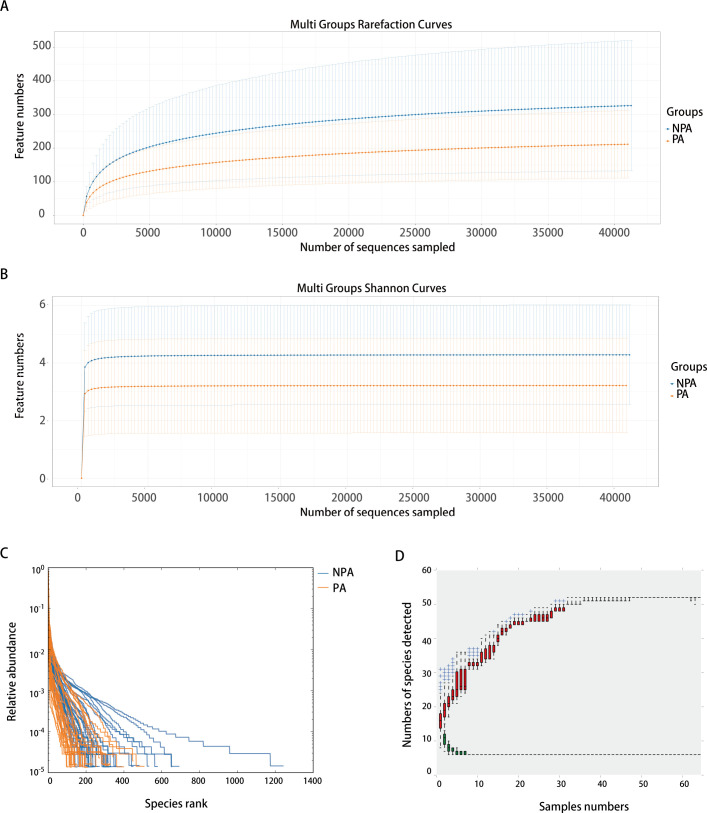
Alpha diversity analysis. (**A**) Rarefaction curve: reflects the change in the number of ASVs as sequencing depth increases. (**B**) Shannon curve (Shannon Index): reflects the microbial diversity of each sample under different sequencing quantities; the x-axis denotes the number of randomly sampled sequencing reads, and the y-axis denotes the Shannon index. (**C**) Rank abundance curve: elucidates two facets of sample diversity, “species richness” and “species evenness”; the x-axis represents the ASV rank, and the y-axis represents the relative percentage of sequence numbers in ASVs at this rank. (**D**) Species accumulation curve: measures and predicts the increment of species richness in the community with the increase in sample size; the x-axis is the sample size, and the y-axis is the number of species after sampling; wherein the red box line indicates the species accumulation curve, and the green box line indicates the species co-occurrence curve.

To validate these findings and ensure the robustness of our sequencing depth, we performed abundance curve analyses (Fig. 3A through D). Both rarefaction and Shannon curves reached a plateau, confirming that the sequencing depth was sufficient to capture the full extent of community diversity. Consistent with the index-based analysis, the NPA group maintained higher species richness and Shannon diversity. The rank abundance curve further illustrated a fundamental shift in community structure: the NPA group not only possessed more high-abundance taxa but also retained a greater diversity of low-abundance taxa, resulting in a more even distribution of species. Additionally, sample accumulation curves confirmed that our sampling strategy was robust enough to capture the vast majority of taxa within the community.

#### Distinct airway microbial community structures associated with *P. aeruginosa* colonization

**Fig 4:**
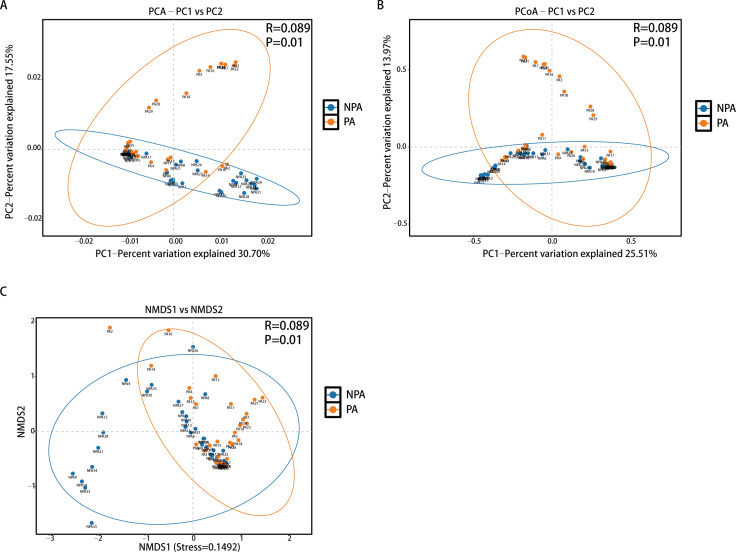
Beta diversity analysis. (**A**) PCA, (**B**) PCoA, and (**C**) NMDS. In the figure, points represent individual samples, different colors denote different groups, and the distance between points reflects the degree of difference. For samples on the coordinate plot, the closer the distance between two samples, the higher their similarity; the farther the distance, the greater the degree of difference.

Analyzing beta diversity revealed distinct microbial community structures between NCFB patients with and without *P. aeruginosa* colonization. Using principal component analysis (PCA), principal coordinate analysis (PCoA), and non-metric multidimensional scaling (NMDS), we characterized the compositional variation within the airway microbiome (Fig. 4A through C). Although partial overlap was observed, samples from the PA and NPA groups exhibited a clear separation trend across all ordination methods. This separation was quantitatively supported by PC1 and PC2, which explained 30.70% and 17.55% of the variance in PCA and 25.51% and 13.97% in PCoA, respectively. The NMDS analysis yielded a stress value of 0.1492, confirming the ordination’s reliability.

Statistical validation confirmed that these structural differences were significant. Permutational tests performed on all three models demonstrated a significant correlation between microbial community composition and *P. aeruginosa* colonization status (correlation coefficient R = 0.089; *P* = 0.01). These findings indicate that while individual patient microbiomes retain unique features, *P. aeruginosa* colonization is a major determinant of community architecture, driving a systematic shift in the airway microbiota that distinguishes colonized patients from their non-colonized counterparts.

#### Colonization by *P. aeruginosa* causes a decrease in the richness of other microbial communities

**Fig 5:**
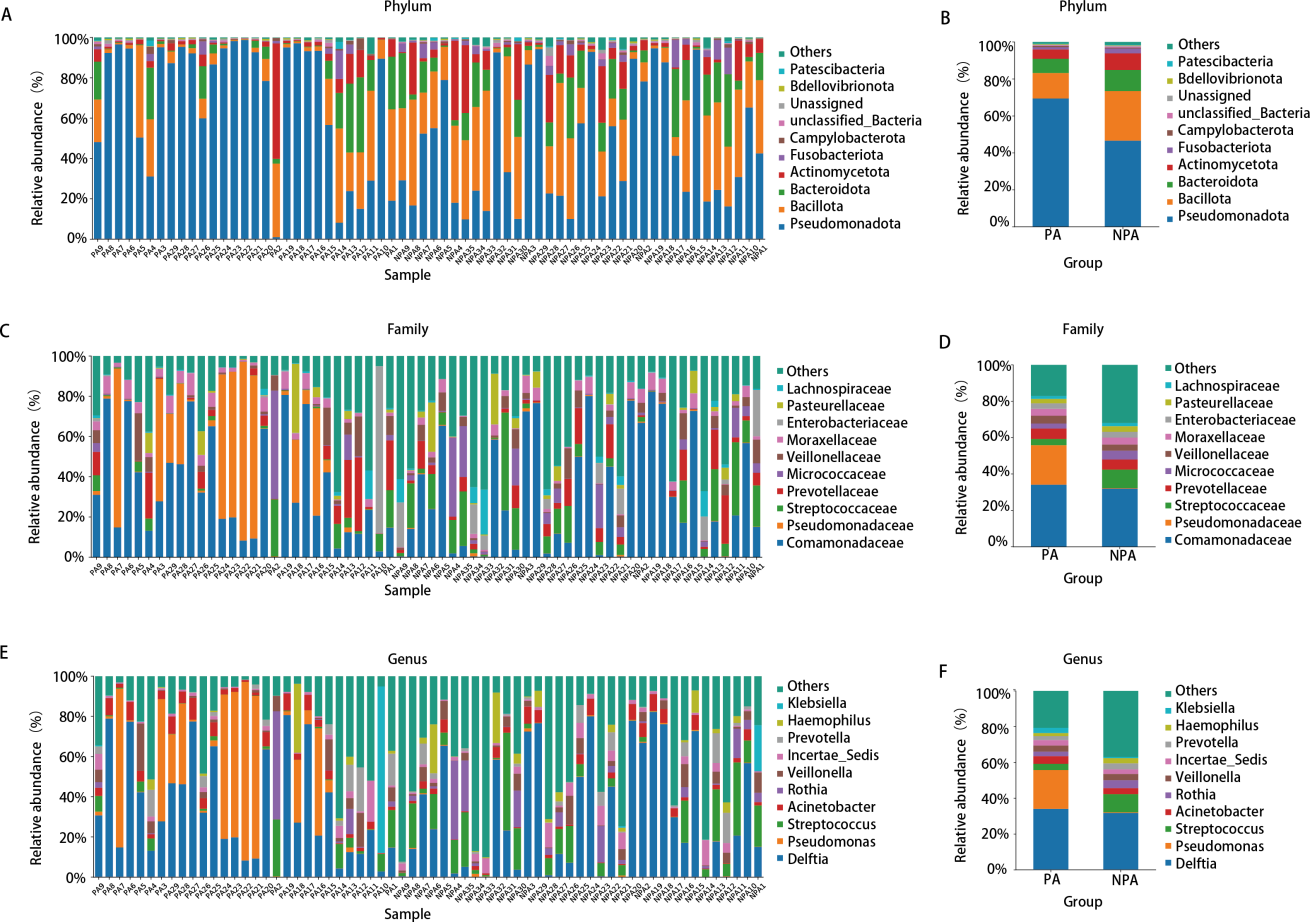
Species distribution of BALF in the *P. aeruginosa* colonization group and non-colonization group. Panels **A–F** are bar charts of species at the phylum (**A, B**), family (**C, D**), and genus (**E, F**) levels in bronchoalveolar lavage fluid samples: the length of color blocks represents the relative abundance proportion of species, the horizontal axis indicates sample groups, and the vertical axis indicates the percentage of relative abundance; each sub-figure displays the top 10 taxonomic groups with relative abundance at the corresponding taxonomic level, and low-abundance taxa are merged into “Others.”

To characterize the structural shifts in the airway microbiome associated with *P. aeruginosa* colonization, we compared the relative abundances of dominant taxa between the PA and NPA groups, BALF of NCFB across taxonomic ranks (Fig. 5A through F). At the phylum level, the microbial landscape was dominated by *Pseudomonadota*, although its abundance was notably lower in the PA group (70.2%) compared to the NPA group (47.5%) (Fig. 5B). Conversely, the NPA group exhibited significantly higher enrichment of commensal phyla, including *Bacillota*, *Bacteroidota*, *Actinomycetota*, and *Fusobacteriota*, suggesting a more diverse and balanced community structure in the absence of *P. aeruginosa*.

This pattern of differential abundance persisted at finer taxonomic resolutions. At the family level, *Pseudomonadaceae* was the predominant taxon in the PA group, accounting for 26.2% of the community, but was nearly absent in the NPA group (0.4%) (Fig. 5D). In contrast, families typically associated with the oral cavity and upper respiratory tract, such as *Streptococcaceae* and *Micrococcaceae*, were markedly expanded in the NPA group.

At the genus level, the ecological dominance of *Pseudomonas* was unequivocal in the PA group (27.3%), whereas it constituted only a negligible fraction (0.5%) in the NPA group (Fig. 5F). The displacement of *Pseudomonas* in the NPA group coincided with a significant rise in a consortium of genera, including *Streptococcus*, *Acinetobacter*, *Rothia*, *Veillonella*, and *Prevotella*. This compositional shift indicates that *P. aeruginosa* colonization is associated with a profound simplification of the microbial ecosystem.

#### Ecological impact and taxonomic resolution of the *Pseudomonas* genus

By analyzing the community changes after removing all *P. aeruginosa* ASV from the data set, the ecological impact of *P. aeruginosa* colonization was assessed. The exclusion of this species led to a significant convergence of alpha diversity indices between the PA and NPA groups (Fig. S2A through E). Before removal, the Shannon index (*P* = 0.0167) and PD_whole_tree index (*P* = 0.0247) showed significant differences between groups (Fig. 2C and E); however, after removal, these differences were not statistically significant (Shannon *P* = 0.0637; PD_whole_tree *P* = 0.315). Although other diversity indices retained statistical significance, the overall trend indicated a substantial narrowing of diversity gaps. Meanwhile, beta diversity clustering was noticeably weakened (Fig. S3A through C), suggesting that the genus *P. aeruginosa* is the main driver of the observed ecological differentiation.

At the same time, corresponding changes in the taxonomic composition were also observed. At the phylum level, the relative abundance of *Pseudomonadota* in the PA group decreased from 70.2% to 65.9%, and in the NPA group from 47.5% to 46.6%, which occurred after the removal of *P. aeruginosa* (Fig. 5B). At a finer taxonomic level, the loss of *P. aeruginosa* ASV allowed previously masked taxa to emerge, resulting in a more even distribution of symbiotic genera between the two groups, such as *Streptococcus*, *Acinetobacter*, *Rothia*, *Veillonella*, and *Prevotella* (Fig. S4A through C).

Refined classification within the genus *Pseudomonas* shows that, in addition to detecting *P. aeruginosa* itself, which accounts for 67.7% of species-level sequencing reads, other non-*aeruginosa* species, such as *Pseudomonas putida*, *Pseudomonas fluorescens*, and *Pseudomonas delhiensis*, were also detected. Additionally, a portion of sequences (4.8%) remained unclassified at the species level. In the NPA group, the genus *Pseudomonas* accounted for only 0.5% of total genus-level reads. Among these *Pseudomonas*-assigned reads, *P. aeruginosa* was nearly absent, representing only 0.2%, whereas most reads were assigned to non*-aeruginosa Pseudomonas* species, predominantly *Pseudomonas putida* (Fig. S4D). These findings confirm the dominant position of *P. aeruginosa* in colonized airways but also indicate that within the genus *Pseudomonas*, there are other non*-aeruginosa Pseudomonas* and unclassified *Pseudomonas* in addition to *P. aeruginosa*.

## DISCUSSION

NCFB is a progressive respiratory disease characterized by chronic cough with sputum, shortness of breath, and recurrent respiratory infections, marked by permanent bronchial dilation, mucus retention, and impaired ciliary clearance function (2). According to a European prospective study, only 60% of NCFB patients have a clear cause. The five most common causes are post-infection (20%), COPD-related (15%), connective tissue disease-related (10%), immunodeficiency-related (5.8%), and asthma-related (27). Colonization with *P. aeruginosa* is a key prognostic predictor in NCFB patients. This study, through the integrated analysis of clinical phenotype stratification and 16S rRNA gene sequencing of BALF, reveals that *P. aeruginosa* colonization is a marker of infection status.

In the patient population with NCFB, whether *P. aeruginosa* colonization occurs has become a focal point of interest in both clinical and basic research on disease progression. This study analyzed clinical information and found significant heterogeneity between patients colonized with the PA group and the NPA group: in the PA group, the proportion of patients with moderate to severe conditions was significantly higher than that in the NPA group in terms of BSI, imaging scores (Bhalla score), and comprehensive functional scores (E-FACED score), consistent with previous research results (28). This finding directly suggests a close association between *P. aeruginosa* colonization and disease severity, whereby colonized patients not only have more severe conditions but also experience significantly increased frequency and duration of hospitalizations, leading to higher consumption of healthcare resources, such as increased use of antimicrobial drugs and total hospitalization costs (29, 30). Patients identified with *P. aeruginosa* colonization may have a higher risk of disease and require more medical care and resource investment. The diagnosis and treatment of such patients need close attention, as *P. aeruginosa* colonization has become one of the important indicators in the progression of NCFB.

*P. aeruginosa* and *Haemophilus* influenzae are common microorganisms in NCFB and play a role in the pathogenesis of airway infections (31). At the same time, these primarily colonizing microbial communities are in a competitive relationship with other microorganisms in the airway environment, affecting the survival of other microorganisms and altering the distribution of microbial communities in the airway environment (32). This study found, through comparisons of microbial diversity and abundance, that colonization by *P. aeruginosa* influences the richness of patients’ airway microbiota. In the *P. aeruginosa* colonization group, the patients’ airway microbiota was predominantly composed of *Pseudomonas*, whereas in the non-*Pseudomonas* colonization group, the abundance of non*-Pseudomonas* genera, such as *Streptococcus* and *Prevotella*, was elevated. These findings suggest that PA colonization disrupts the microbial environmental balance in the airways, initiating a vicious cycle wherein *P. aeruginosa* colonization leads to dysbiosis, which subsequently induces airway inflammation and structural destruction, ultimately driving the progression of NCFB. Clinical data analysis suggests that patients colonized with *P. aeruginosa* have more severe disease, which may be related to the imbalance of the airway microbiome following *P. aeruginosa* colonization. Existing studies have shown that microbial dysbiosis can affect the progression of various diseases (33, 34); for example, intestinal microbiota imbalance is involved in the progression of Alzheimer’s disease (35), and regulating the gut microbiota can aid in the treatment of diabetes (36). These findings indicate the key role of microbial distribution in diseases. However, whether airway microbiota dysbiosis similarly affects the progression of lung diseases remains unclear. Therefore, restoring the disrupted microbial environment caused by *P. aeruginosa* colonization may be a therapeutic strategy to reduce disease severity in patients colonized with *P. aeruginosa*.

*P. aeruginosa* colonization is a critical driving factor in the disease progression of patients with NCFB. This study found that *P. aeruginosa* colonization significantly increases the risk of acute exacerbations and the annual number of hospitalizations in NCFB patients. It is positively correlated with disease severity (BSI, Bhalla, E-FACED scores) and inflammatory markers (peripheral blood leukocyte count, neutrophil percentage), and the difference in the proportion of moderate to severe cases according to the BSI score is statistically significant (*P* < 0.001). Although the continuous levels of systemic inflammatory markers did not reach statistical significance between groups, we observed numerically higher proportions of elevated WBC counts (31% vs 17.1%) and neutrophil percentages (41.4% vs 31.4%) in NCFB patients with *P. aeruginosa* colonization. These descriptive trends suggest a potential clinical signal of heightened inflammation in colonized individuals. However, the lack of statistical significance likely indicates that the present study was underpowered to detect subtle systemic inflammatory changes, possibly due to sample size limitations or phenotypic heterogeneity within the colonized group. Therefore, larger, multicenter prospective cohorts are warranted to definitively elucidate the impact of *P. aeruginosa* colonization on systemic inflammation.

At the microbiome level, PA colonization significantly reduces the alpha diversity of BALF microbiota, with abundance dominated by *Pseudomonas*. While the lower alpha diversity observed in the PA group might traditionally be interpreted as a sign of ecological disruption, we acknowledge that this reduction is at least partly driven by the compositional dominance of *P*. aeruginosa itself. Given its high relative abundance in these samples, this “compositional effect” naturally compresses the proportional representation of co-existing taxa, which may not necessarily reflect their absolute eradication or active suppression. Perhaps it also has some effect on the growth of symbiotic bacteria, such as *Prevotella*, *Porphyromonas*, and *Streptococcus*, leading to airway microecological imbalance. *P. aeruginosa* colonization status can serve as an important biomarker for high-risk stratification of NCFB (e.g., moderate to severe patients) and for targeted interventions (e.g., anti-colonization and microbiota restoration). While we acknowledge the caution raised by Dickson et al. (37) regarding the potential for high pathogen loads to mask low-abundance taxa in 16S rRNA sequencing, our data support a biological basis for the observed diversity loss. The fact that removal of *P. aeruginosa* ASV led to a convergence of diversity metrics between groups suggests that the high abundance of *Pseudomonas* is not merely obscuring other taxa, but is associated with their actual depletion or exclusion from the niche. This aligns with the concept of *Pseudomonas*-driven dysbiosis, rather than a technical sampling bias.

The sensitivity analysis, which removed all *P. aeruginosa* ASVs, provided critical insight into the nature of airway dysbiosis during *P. aeruginosa* colonization of NCFB patients. Excluding this species led to a marked convergence of diversity metrics between the PA and NPA groups. Furthermore, the distinct clustering of samples based on beta diversity was largely attenuated (Fig. S4A through C). These findings strongly suggest that the high relative abundance of *P. aeruginosa* does not simply overlay a pre-existing diverse community. Instead, it acts as a dominant ecological filter, actively restructuring the underlying microbial landscape. By occupying a large proportion of the niche space, *P. aeruginosa* compresses the relative abundance of co-existing taxa, leading to the observed reduction in diversity and a simplified microbiome structure.

A further detailed analysis of the genus *P. aeruginosa* revealed that, although *P. aeruginosa* is the main species in the PA group of the genus *Pseudomonas,* non*-Pseudomonas aeruginosa* and unclassified *Pseudomonas* were also detected (Fig. S4D). This finding suggests that there are more complex multi-microbial dynamics within this genus.

This study has several limitations that warrant consideration. First, the current single-center, retrospective design limits the generalizability of our findings. The correlation between dynamic changes in NCFB patients with *P. aeruginosa* colonization and clinical outcomes needs to be validated in multicenter prospective cohorts. Furthermore, defining airway colonization based on a single positive culture may have introduced heterogeneity, potentially capturing cases of transient carriage rather than established chronic colonization. This heterogeneity within the colonized group might have diluted observed differences in clinical outcomes (e.g., hospitalization rates) and could explain why some differences did not reach statistical significance. Additionally, while 16S rRNA sequencing provided insights into microbial composition, it lacks functional information. Integrating metagenomic sequencing (mNGS) with metabolomic analysis would reveal specific functional changes associated with *P. aeruginosa* colonization, such as virulence factor synthesis and the spread of antibiotic resistance genes, thereby providing more precise targets for mechanistic research.

### Conclusion

This study indicates that colonization by *P. aeruginosa* increases the risk of acute exacerbations and hospitalization, and is positively correlated with disease severity and inflammatory markers. *P. aeruginosa* colonization leads to a decrease in the α-diversity of the bronchoalveolar lavage fluid microbiota, dominance of the *Pseudomonas* genus, and suppression of commensal bacteria, thereby causing microecological imbalance.

## Data Availability

The data sets used during the study are available in the Sequence Read Archive (SRA) at PRJNA1438598.

## SUPPLEMENTAL MATERIAL

Supplemental Figures spectrum.00871-26-s0001.docx Fig. S1 to S4.

ASM does not own the copyrights to Supplemental Material that may be linked to, or accessed through, an article. The authors have granted ASM a non-exclusive, world-wide license to publish the Supplemental Material files. Please contact the corresponding author directly for reuse.
