# Supplementary material for: Analysis of clinical characteristics and bronchoalveolar lavage fluid microbial community diversity in non-cystic fibrosis bronchiectasis patients with Pseudomonas aeruginosa colonization
Source: Microbiol Spectr. 2026 Jun 23;14(8):e00871-26. doi: 10.1128/spectrum.00871-26 (PMC13436378; doi:10.1128/spectrum.00871-26)
Supplement: Supplementary file 1 [file spectrum.00871-26-s0001.docx]

**Supplementary Figure S1. (A)** LabChip fragment analysis of sequencing libraries. Lower Marker (LM, ~ 1 bp) and Upper Marker (UM, ~ 10,000 bp) served as internal size standards. No amplified products were detected in the kit-only negative control between LM and UM, ruling out reagent contamination. All patient samples showed specific peaks within the expected size range. (B) Amplicon size distribution and summary of sequencing reads. The bar chart shows the total sequencing reads for the *P. aeruginosa* colonized group (PA, purple) and the non-colonized group (NPA, blue) in different amplicon length ranges.

**Supplementary Figure S2.** Analysis of Alpha diversity indices after deleting the ASV of *P. aeruginosa* (A-E); ACE: Abundance‐based Coverage Estimator, an index estimating the total number of species based on the species accumulation curve; Chao1: A community species richness index based on ASVs; Shannon: A comprehensive diversity index reflecting both species richness and evenness; Simpson: A diversity index reflecting the proportion of dominant species; PD_whole_tree: A community phylogenetic diversity index based on the phylogenetic tree. Note: The horizontal axis represents different groups, and the vertical axis represents the Alpha diversity index values. The Wilcoxon test was used, and *P* < 0.05 indicates a statistically significant difference.

**Supplementary Figure S3.** Beta diversity analysis after deleting the ASV of *P. aeruginosa*. (A) Principal component analysis (PCA). (B) Principal coordinates analysis (PCoA). (C) Non-metric multidimensional scaling (NMDS). In the figure, points represent individual samples, different colors denote different groups, and the distance between points reflects the degree of difference. On a coordinate plot, the closer the distance between two samples, the greater their similarity; the farther apart, the greater the difference.

**Supplementary Figure S4.** Impact of *Pseudomonas* removal and intrageneric species distribution. (A-C) are bar charts of species at the phylum (A), family(B), and genus(C) levels in bronchoalveolar lavage fluid samples: the length of color blocks represents the relative abundance proportion of species, the horizontal axis indicates sample groups, and the vertical axis indicates the percentage of relative abundance; each sub-figure displays the top 10 taxonomic groups with relative abundance at the corresponding taxonomic level, and low-abundance taxa are merged into “Others”. (D) Perform species classification within the genus *Pseudomonas*.
